# Gossypol Exhibited Higher Detrimental Effect on Ruminal Fermentation Characteristics of Low-Forage in Comparison with High-Forage Mixed Feeds

**DOI:** 10.3390/toxics9030051

**Published:** 2021-03-08

**Authors:** Wei-Kang Wang, Yan-Lu Wang, Wen-Juan Li, Qi-Chao Wu, Sheng-Li Li, Hong-Jian Yang

**Affiliations:** State Key Laboratory of Animal Nutrition, College of Animal Science and Technology, China Agricultural University, Beijing 100193, China; 18292092306@163.com (W.-K.W.); wang_yanlu@cau.edu.cn (Y.-L.W.); liwjuan1226@163.com (W.-J.L.); wuqichao@cau.edu.cn (Q.-C.W.); lisheng0677@163.com (S.-L.L.)

**Keywords:** gossypol, in vitro fermentation, rumen microbes

## Abstract

Gossypol is a key anti-nutritional factor which limits the feeding application of cottonseed by-products in animal production. A 2 × 4 factorial in vitro experiment was conducted to determine the effect of gossypol addition levels of 0, 0.25, 0.5, and 0.75 mg/g on ruminal fermentation of a high-forage feed (HF, Chinese wildrye hay/corn meal = 3:2) in comparison with a low-forage feed (LF, Chinese wildrye hay/corn meal = 2:3). After 48 h of incubation, in vitro dry matter disappearance was greater in the LF than the HF group, while the cumulative gas production and asymptotic gas production were greater in the HF than the LF group (*p* < 0.05). Regardless of whatever ration type was incubated, the increasing gossypol addition did not alter in vitro dry matter disappearance. The asymptotic gas production, cumulative gas production, molar percentage of CO_2_ and H_2_ in fermentation gases, and microbial protein in cultural fluids decreased with the increase in the gossypol addition. Conversely, the gossypol addition increased the molar percentage of CH_4_, ammonia N, and total volatile fatty acid production. More than 95% of the gossypol addition disappeared after 48 h of in vitro incubation. Regardless of whatever ration type was incubated, the real-time PCR analysis showed that the gossypol addition decreased the populations of *Fibrobacter* *succinogenes*, *Ruminococcus albus*, *Butyrivibrio fibrisolvens*, *Prevotella ruminicola*, *Selenomonas ruminantium*, and fungi but increased *Ruminococcus flavefaciens*, protozoa, and total bacteria in culture fluids in comparison with the control (*p* < 0.01). Additionally, the tendency of a smaller population was observed for *R. albus*, *B. fibrisolvens*, and fungi with greater inclusion of gossypol, but a greater population was observed for *F. succinogenes*, *R. flavefaciens*, *S. ruminantium*, protozoa, and total bacteria. In summary, the present results suggest that rumen microorganisms indeed presented a high ability to degrade gossypol, but there was an obvious detrimental effect of the gossypol addition on rumen fermentation by decreasing microbial activity when the gossypol inclusion exceeded 0.5 mg/g, and such inhibitory effect was more pronounced in the low-forage than the high-forage group.

## 1. Introduction

Gossypol (C_30_H_30_O_8_), a polyphenolic compound found in the cotton plant (*Gossypium* sp.) [1], is commonly regarded as one of the key anti-nutritional factors that limit the feeding application of cottonseed by-products (e.g., whole cottonseed, cottonseed meal) in animal production. In the animal nutritionist community, adult ruminants with mature microbial fermentation functionality in the rumen are commonly considered more tolerant to gossypol than monogastric animals and young ruminants [2]. For a long time, the current knowledge has believed that the high tolerance to gossypol by adult ruminants should be attributable to microbial detoxification in the rumen via binding to soluble proteins and microbial degradation [3,4]. Clinical signs of high gossypol intake on host animals in terms of erythropoiesis [5,6], milk performance [7], and postpartum estrus in dairy cows [8] and sperm production in bulls [9,10,11] have been well recognized, and the maximal tolerant gossypol intake limit in feeds (<500 mg/kg) has been set for different farm animals in feeding practice in the European Union [12].

The rumen microbiome is a highly diverse collection of obligately anaerobic microbes including fungi, protozoa, bacteria, and archaea [13]. The rumen fermentation response to gossypol exposure has not been well described until now, and it was not clear if the tolerance of rumen microbes to gossypol could differ depending on the forage to concentrate ratio. In the present study, the objective is threefold: firstly, to determine how much gossypol could be degraded; secondly, to investigate if an increasing gossypol addition could inhibit rumen fermentation characteristics; and, finally, to clarify if the detrimental effect could differ depending on the forage level in diets.

## 2. Materials and Methods

### 2.1. Gossypol Solution Preparation

Forty mg of gossypol chemical (>97%, Sigma-Aldrich, Darmstadt, Germany) was dissolved in 2 mL glacial acetic acid to serve as a standard gossypol solution. Prior to the experiment, the solution was diluted with glacial acetic acid to obtain three working solutions containing 5, 10, and 15 mg/mL gossypol, respectively.

### 2.2. Rumen Fluid Preparation

All animal experimental procedures were conducted in accordance with the Institutional Animal Care Committee of China Agricultural University (CAU20171014-1). Three middle lactating Holstein cows (daily milk yield of 20.5 ± 0.9 kg) were used as rumen fluid donors. These rumen-fistulated cows had free access to water and were fed with 12.5 kg total mixed ration with 520 g/kg dry matter (DM) content twice daily at 07:00 a.m. and 05:00 p.m. The ration consisted of 490 g maize silage, 110 g alfalfa hay, 140 g maize meal, 260 g commercial concentrate per kg of DM. Rumen fluid from each cow was collected 2 h after the morning feeding and squeezed through four layers of cheesecloth. The filtrated rumen fluids from the three cows were mixed in equal proportion and filled with CO_2_ at 39 °C in a water bath prior to in vitro inoculation.

### 2.3. Experimental Design and In Vitro Incubation

Following a 2 × 4 factorial in vitro experimental design, a high-forage mixed substrate (HF, Chinese wildrye hay/corn meal—3:2, *w/w*) and a low-forage mixed substrate (LF, Chinese wildrye hay/corn meal—2:3, *w/w*) were prepared in the present study. Afterwards, each of the above substrates was incubated in vitro with gossypol inclusion at 0, 0.25, 0.5, and 0.75 mg/g substrate on DM basis. The chemical composition of two rations is shown in Table 1.

For each substrate, 500 mg substrate was placed into 40 bottles (4 gossypol addition levels × 10 replicates—40 bottles per substrate) with 50 mL buffer solution (pH 6.87) [14] and 25 mL filtered rumen fluid. The gossypol working solutions (25 μL) were added to these bottles, resulting in the three afore mentioned gossypol treatment levels, and 25 μL gossypol-free glacial acetic acid solution was added to the control group for each substrate. All of these bottles were purged with anaerobic N_2_ to obtain an anaerobic condition and sealed with butyl rubber stoppers and Hungate’s screw caps. For each substrate, 20 bottles (4 gossypol addition levels × 5 replicates—20 bottles per substrate) were individually connected to an automated gas production recording system (AGRS-III, China Agricultural University, Beijing, China) [15] and incubated at 39 °C for 48 h. Simultaneously, an additional 20 bottles (4 gossypol addition levels × 5 replicates—20 bottles per substrate) prepared in the same manner were separately connected to pre-emptied air bags and incubated at the same condition as above for later analysis of gas composition.

### 2.4. Sampling and Analysis

#### 2.4.1. Sample Collection

After 48 h of incubation, all bottles were disconnected from the AGRS-III system and air bags. The pH values in culture fluids were measured immediately. The remaining biomasses of each bottle were separately filtered through pre-weighed nylon bags. The bags containing filtrated residues were then rinsed in tap water until the water ran clear, then squeezed by hand to remove excess water, and dried at 65 °C for 24 h for in vitro dry matter disappearance (IVDMD) analysis. One sample of 1 mL culture fluids from each bottle was mixed with 0.3 mL of 250 g/L meta-phosphoric acid solution at 4 °C for 30 min and centrifuged at 10,000× *g* for 15 min at 4 °C. The supernatants were kept at −20 °C for later analysis of volatile fatty acids (VFAs) and ammonia N. Three samples (1 mL × 3) of culture fluids were stored at −20 °C for gossypol and microbial protein (MCP) analysis. Additionally, a sample of 1 mL culture fluids was stored at −80 °C for the analysis of microbial population.

#### 2.4.2. Chemical Analysis and Calculations

Crude protein, ether extract, and starch were determined according to the methods of AOAC [16]. Neutral detergent fiber and acid detergent fiber were determined according to the methods of Van Soest et al. [17]. Ammonia N and MCP concentrations were determined by spectrophotometry according to the methods of Verdouw et al. [18] and Cui et al. [19], respectively. The VFAs concentration and fermentation gas composition were determined by gas chromatography according to the methods of Zhang and Yang [15] and Cui et al. [19], respectively.

Cumulative GP data [GP(t), mL/g DM] at time (t) were fitted to a nonlinear model [20] by iterative regression analysis, as shown in Equation (1):

GP(t) = A/[1 + (C/t)^B^] (1)

where A represents the asymptotic GP generated at a constant fractional rate (c) per unit time, t is the time of the gas recording, B is a sharpness parameter determining the shape of the curve, and C is the time (h) at which half of A is reached. The value of A–C was estimated using the nonlinear procedure of SAS [21]. The average GP rate (AGPR) at half of A was calculated according to Equation (2):

AGPR = (A × B)/(4 × C)(2)

Isobutyrate and isovalerate were summed as branched-chain VFAs (BCVFA). The ratio of non-glucogenic-to-glucogenic acids (NGR) was calculated [22] as Equation (3):

NGR = (acetate + 2 × butyrate + valerate)/(propionate + valerate)(3)

where VFAs were expressed in molar proportion.

Fermentation efficiency (FE) of energy from carbohydrates to VFAs was estimated using Equation (4):

FE = (0.622 × acetate + 1.092 × propionate + 1.56 × butyrate)/(acetate + propionate + 2 × butyrate)(4)

#### 2.4.3. Gossypol Extraction and High-Performance Liquid Chromatography Analysis

Gossypol in rumen fluid samples was extracted according to the method described by Zhong [23]. The contents of gossypol were quantified by high-performance liquid chromatography using a Wufeng analytical instrument (Wufeng Co., Ltd., Shanghai, China) consisting of an LC-P100PLUS pump, LC-UV100PULS UV detector, LC-CO100PLUS column heater, and a symmetry reversed-phase C18 column (250 × 4.6 mm, 5 μm, pH 2–8, Waters, Milford, MA, USA). The method was set as follows: The mobile phase was acetonitrile and 0.2% phosphoric acid solution at a ratio of 85:15 (*v/v*) under a flow rate of 1 mL/min. Injections were 20 μL, and gossypol was detected at 235 nm with an analysis temperature of 25 °C.

**Figure 1:**
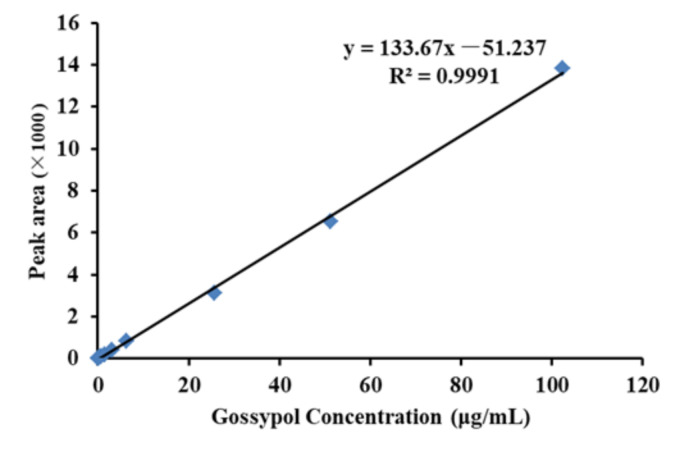
Standard curve of gossypol.

An amount of 100 mg standard gossypol was dissolved into 1 mL mobile phase and diluted to 100, 50, 25, 12.5, 6.25, 3.125, 1.55, 0.78, 0.36, 0.18, and 0.09 μg/mL by mobile phase. The standard curve (Figure 1) was obtained by linear regression of the peak area (Y) and gossypol concentration (X) under optimal chromatographic conditions as noted above. Gossypol standards shown peaked at a retention time of 5 min.

#### 2.4.4. DNA Extraction and Real-Time PCR Amplification of 16S RNA Genes

The populations of protozoa, fungi, total bacteria, and six major bacteria (*Fibrobacter succinogenes*, *Ruminococcus flavefaciens*, *Ruminococcus albus*, *Butyrivibrio fibrisolvens*, *Prevotella ruminicola*, *Selenomonas ruminantium*) were determined by qPCR. The total DNA of the rumen fluid sample was extracted by using the QIAamp^®^ DNA stool mini kit (Qiagen, Hilden, Germany) following the manufacturer’s instructions. The concentration of total DNA from each sample was estimated using a Nanodrop spectrophotometer 2000 (Thermo Scientific, Wilmington, DE, USA) at 260 nm.

Quantitative PCR was conducted with ABI 7500 FAST real time PCR system (ABI 7500, Applied Biosystems, Foster City, CA, USA) using TB Green Premix Ex Taq II (Tli RNaseH Plus) (Takara, Dalian, Liaoning, China). The total volume of amplification reactions was 50 μL, including 4 μL of template DNA, 25 μL of TB Green Premix Ex Taq II (Tli RNaseH Plus) (2×), 1 μL of ROX Reference Dye (50×), 2 μL of each primer (10 pmol/μL), as shown in Table 1, and 16 μL sterile H_2_O. The cycling conditions contained the following: an initial denaturation step at 95 °C for 30 s, followed by 40 cycles of 95 °C for 3 s and 60 °C for 30 s. Dissociation curve of PCR end products was used for judging the amplification specificity. All PCRs were performed in triplicate. The normalized fluorescence data were converted to a log scale and the threshold cycle value was calculated by determining the threshold (Ct; the cycle at which the threshold line crosses the amplification curve).

#### 2.4.5. Preparation of Standard Plasmid for Real-Time PCR

The target DNA was amplified by absolute quantification PCR using plasmid DNA containing the respective target gene sequence as standard DNA, and the species-specific primer sets used are shown in Table 2. The PCR product sizes were confirmed by agarose gel and purified using the QIAquick gel extraction kit (Qiagen, Valencia, CA, USA). Then, the PCR products were ligated into pTZ57RT cloning vector (Fermentas, Berlin, Germany), and we transformed the ligated products into competent *Escherichia coli* DH5 alpha cells by heat shock. The plasmids were purified from positive clones and confirmed by PCR amplification with the respective primer sets. The concentration of the target DNA was determined by Nanodrop spectrophotometer 2000 (Thermo Scientific, Wilmington, USA). Copy number per μL of standard plasmid was calculated based on the formula: Copy No/μL = Concentration of plasmids (g/μL) × 6.022 × 10^23^ / length of recombinant plasmid (bp) × 660, (660 = Molecular weight of one basepair, 6.022 × 10^23^ = Avogadro’s number). The amplification of each ample as well as the 10-fold dilution series of the standard plasmid ranging from 10^11^ to 10^5^ copies for the respective target DNA was run in triplicate.

### 2.5. Statistical Analysis

All the experimental data, including the interaction effect of rations and gossypol addition levels, were analyzed by using the general linear model procedure of SAS (1999) with a multiple comparison test (Tukey/Kramer). The linear and quadratic polynomial contrasts were used to examine responses of different rations for increasing gossypol addition levels by ANOVA. Significance was declared at *p* < 0.05 unless otherwise noted.

## 3. Results and Discussion

### 3.1. Effect of Gossypol on IVDMD and Kinetic Gas Production

In the present study, two rations used were designed to simulate the forage to concentrate ratios of cows at different stages of lactation, and the purpose of the four gossypol addition levels used was to study the effect of gossypol concentrations below or above the safety limit (500 mg/kg) on rumen fermentation characteristics and the population of rumen microbes. As shown in Table 3, a greater IVDMD and proportion of CH_4_ occurred in LF (*p* < 0.01), while total cumulative gas production, asymptotic gas production, and the proportion of CO_2_ were greater in HF. Regardless of whatever ration type was incubated, IVDMD, total cumulative gas production, asymptotic gas production, and the proportion of CO_2_ and H_2_ decreased against the gossypol addition, but the proportion of CH_4_ increased with the augmentation of the gossypol addition dose from 0 to 0.5 mg/g.

The soluble and easily degraded components in feeds are always utilized by rumen microbes first, followed by the insoluble but potentially digestible components. The greater IVDMD in LF than HF could be due to the degradation characteristics of ration components in the present study. Although the ration type presented a greater effect on IVDMD than the gossypol addition dose, the cumulative gas production, asymptotic gas production, and the proportion of CO_2_ and H_2_ showed a significant numeric decrease with the increase in the gossypol addition dose, and it noted that there was an obvious inhibitory effect of gossypol on rumen fermentation. The volume of cumulative gas production was positively correlated with dry matter degradability, but the current results show that cumulative gas production of HF was greater than LF, suggesting that gossypol presented a greater inhibitory effect on LF than HF, and we speculated this could be due to the higher tolerance of rumen microbes in HF to gossypol. The results of the present study evidence that inclusion of gossypol could increase CH_4_ emissions which has not been reported before, and the promoting effect of gossypol on CH_4_ was more obvious in the LF group. The main pathway of CH_4_ synthesis in the rumen is reduction in CO_2_ and H_2_ by methanogens, which may explain the increase in CH_4_ with the decrease in CO_2_ and H_2_ when the gossypol addition dose was less than 0.5 mg/g. The increase in methane production suggests that an appropriate amount of gossypol could promote the activity of methanogens and improve the utilization efficiency of CO_2_ and H_2_, but the mechanism is still unclear.

### 3.2. Effect of Gossypol on In Vitro Rumen Fermentation Characteristics

As shown in Table 4, pH and ammonia N were higher for HF than LF (*p* < 0.01), and the values increased with the augmentation of the gossypol addition dose. The concentrate of MCP was higher for HF than LF and decreased linearly with the increase in the gossypol dose. Total VFAs and the molar proportion of acetate and BCVFA were greater for HF than LF, and the values increased with the augmentation of gossypol from 0.25 to 0.5 mg/g. The proportion of butyrate and NGR were not affected by the type of ration, and the proportion of valerate and FE were not affected by ration type or the increase in the gossypol addition dose. Total disappearance of gossypol was higher in HF (*p* < 0.01), and it showed an obvious increase trend when gossypol increased from 0.25 to 0.5 mg/g but decreased significantly when gossypol was at 0.75 mg/g (*p* < 0.01). Total disappearance of gossypol in all addition treatments could reach over 95% after 48 h of incubation.

The optimal rumen pH plays a key role in the maintenance of normal rumen function, and the appropriate pH in rumen liquid for ruminal microbes ranged from 6.6 to 6.8 [29]. In the present study, pH values ranged from 6.61 to 6.68 and were within the acceptable limits. The concentration of ammonia N is an essential indicator of the N-metabolism level in the rumen. As an intermediate product of rumen fermentation, ammonia N is either the nitrogen source of MCP synthesis or the end product of dietary protein metabolism. The ammonia N concentration in the present study increased along with the augmentation of the gossypol addition dose, but MCP linearly decreased against the gossypol addition (*p* < 0.01), suggesting that the conversion efficiency of ammonia N to MCP was affected by the detrimental effect of gossypol on rumen microbes.

The VFAs are end products of rumen fermentation which provide the main metabolizable energy resource for host ruminants [30]. Total VFAs concentration was greater in the HF than the LF group in the present study, depending on the difference in the nutrient density in the rations. Total VFAs production of both rations showed a numerical increase with the augmentation of gossypol from 0 to 0.5 mg/g, which confirmed that the gossypol addition dose below 0.5 mg/g could promote the rumen fermentation efficiency. About 80% maintenance energy of ruminants was provided by acetate, propionate, and butyrate [31]. Acetate is an essential VFA for the synthesis of milk fat, while propionate is an important VFA for the synthesis of milk lactose [32]. The greater acetate concentration in HF than LF was due to the greater content of fiber in HF, suggesting the fermentation pattern of the HF ration belonged to acetate fermentation. The greater propionate concentration in LF than HF was due to the dominant position of the soluble carbohydrate (e.g., starch) fermentation of LF, suggesting the fermentation pattern of the LF ration belonged to propionate fermentation. Regardless of whatever ration type was incubated, the molar proportion of propionate decreased while molar proportions of acetate and butyrate increased in the gossypol addition group in comparison with the control. The present study observed an inhibitory effect of the gossypol addition on the activity of ruminal lactate-utilizing bacteria, such as *S. ruminantium* which plays a key role in the conversion of lactate to propionate. The molar propionate proportion showed a significant decrease when the gossypol addition dose increased from 0.25 to 0.75 mg/g, suggesting that there could be a detrimental effect of the gossypol addition on milk production by providing less glucogenic energy for the formation of milk lactose. Moss et al. [33] found that there was a positive relationship between methane production and the molar portion of acetate and butyrate due to a competition existing between propionate formation and the synthesis of methane from hydrogen in the rumen. The decrease in propionate and the increase in acetate and butyrate proportions were associated with the increase in methane production in the present study. Until now, there has been no report about the methane-promoting effect of gossypol, and further animal trials are necessary in the near future to be conducted to confirm the present in vitro results. The total VFAs and disappearance of gossypol decreased significantly when gossypol was at 0.75 mg/g, suggesting that there was an obvious inhibitory effect of gossypol on rumen fermentation when it exceeded the safety limit amount of the European Union (500 mg/kg). In addition, the total disappearance of gossypol was higher in HF than LF, suggesting that gossypol degradation could relate to the process of fiber degradation, and the ration with a high portion of forage was more conducive to the degradation and transformation of gossypol by rumen microbes.

### 3.3. Effect of Gossypol on Rumen Microbial Population

*F. succinogenes*, *R. flavefaciens,* and *R. albus* are the main fibrolytic bacteria in the rumen [34]. As shown in Table 5, the population sizes of target fibrolytic bacteria in the rumen fluid were greater in HF than LF and ranked as *R. flavefaciens* > *F. succinogenes* > *R. albus*. According to Michalet-Doreau et al. [35,36] and Koike et al. [37], *F. succinogenes* was the most dominant species among the three cellulolytic bacteria, whereas *R. albus* was the least abundant species, which is consistent with results in the present study. Compared with the control, the supplementation of rations with gossypol decreased the population of *F. succinogenes* and *R. albus* (*p < 0.01*), while the population of *R. flavefaciens* was positively influenced by the addition of gossypol, implicating that fibrolytic bacteria might show different tolerances to gossypol, and gossypol was not harmful to all microbes in the rumen. In addition, the population of *F. succinogenes* and *R. flavefaciens* showed a numeric increase with the increase in the gossypol addition dose, and this likely implicates that there could be a promoting effect of gossypol on the population of *F. succinogenes* and *R. flavefaciens* by utilizing gossypol and its degradation products as growth resources.

The population of *B. fibrisolvens* and *P. ruminicola* in the present study was significantly higher in LF than HF. Both *Butyrivibrio* and *Prevotella* sp. in the present study represented considerable amounts of bacteria in the two types of ration, suggesting there was a great deal of metabolic diversity in these genera. Most ruminal *Butyrivibrio* genera play a key role in the degradation of starch and pectins and can use urea and ammonia as nitrogen sources [38]. However, *B. fibrisolvens* in the present study seems to be capable of utilizing not only starch and protein but also cellulose. *P. ruminicola* plays a key role in ruminal protein degradation and some strains can ferment xylan, pectin, and starch instead of cellulose. The population of *B. fibrisolvens* and *P. ruminicola* had an obvious decrease with the addition of gossypol (*p* < 0.01), and it corresponded to the decrease in the concentration of ammonia N in comparison with the control, likely suggesting there was a detrimental effect of gossypol on the population of proteolytic bacteria.

*S. ruminantium* has been found to utilize a wide range of substrates, and it plays a key role in the synthesis of propionate, malate, and lactate from primary fermentation products such as pyruvate and succinate in the rumen [39,40]. Hence, the higher population of *S. ruminantium* in the LF group could participate in the utilization of lactic acid produced within rumen fermentation, and the increasing *S. ruminantium* populations with the increase in the gossypol addition dose from 0.25 to 0.5 mg/g could relate to the decrease in gossypol by utilization.

**Figure 2:**
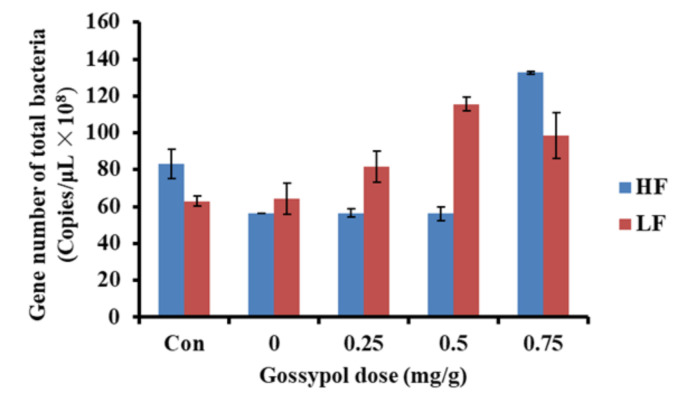
Interaction effect of gossypol dose and ration on the population of total bacteria (HF, Chinese wildrye hay/corn meal—3:2; LF, Chinese wildrye hay/corn meal—2:3).

Both protozoa and total bacteria increased linearly with the augmentation of the gossypol addition dose, whereas the population of fungi decreased significantly. This suggests that fungi were more sensitive to gossypol than bacteria and protozoa, and there could be a detrimental effect of gossypol on the growth and reproduction of fungi. Additionally, protozoa can secret enzymes which degrade the cell walls of fungi [41], and they compete with fungi for nutrients [42]. Consequently, the increase in protozoa could also inhibit the number of fungi. In the present study, there was an interaction effect of the ration and gossypol on the population of total bacteria, as shown in Figure 2. The total bacteria population of LF increased significantly when the gossypol addition level reached 0.75 mg/g, while that of the HF group decreased obviously. This suggests that the detrimental effect of gossypol on rumen bacteria was more obvious on the LF ration than HF when the gossypol addition dose exceeded the safety limit. We speculate that the bacteria of the LF group survived with the greater degradation and transformation ability of gossypol, and then alleviated the toxicity of the excess gossypol.

In present study, the population of both fibrolytic and amylolytic bacteria showed a trend towards increased levels with the increase in the gossypol addition dose from 0.25 to 0.5 mg/g, suggesting that energy metabolism could play an essential role in the process of gossypol detoxification by rumen bacteria, and this was also demonstrated by the previous results of Yang et al. [43].

## 4. Conclusions

Except for the ration type, the addition of gossypol affected in vitro fermentation characteristics in terms of reduced cumulative gas production and microbial protein, but it increased ammonia N and VFA concentrations. The gossypol addition increased the population of rumen bacteria responsible for fiber and starch metabolism. Although mixed rumen microbes showed a high ability to degrade gossypol (>0.95), there was an obvious detrimental effect of gossypol on the activity of rumen microbes and rumen fermentation when the gossypol inclusion exceeded 0.5 mg/g in rations, and such inhibitory effect was more pronounced on the low-forage than the high-forage ration.

## Figures and Tables

**Table 1:** Chemical composition of two substrates (%, DM).

Item ^1^	HF	LF
Crude protein	7.5	8.0
Neutral detergent fiber	45.0	33.0
Acid detergent fiber	25.6	18.4
Ether extract	3.5	4.0
Starch	28.4	41.2

^1^ HF, high-forage substrate; LF, low-forage substrate.

**Table 2:** Primers for real-time PCR.

Target Groups	Sequence 5′–3′	Product Size (bp)	References
*F. succinogenes*	F: GTTCGGAATTACTGGGCGTAAAR: CGCCTGCCCCTGAACTATC	121	[24]
*R. flavefaciens*	F:CGAACGGAGATAATTTGAGTTTACTTAGGR: CGGTCTCTGTATGTTATGAGGTATTACC	132	[24]
*R. albus*	F: CCCTAAAAGCAGTCTTAGTTCGR: CCTCCTTGCGGTTAGAACA	175	[25]
*B. fibrisolvens*	F:TAACATGAGAGTTTGATCCTGGCTCR:CGTTACTCACCCGTCCGC	136	[26]
*P. ruminicola*	F:GAAAGTCGGATTAATGCTCTATGTTGR:CATCCTATAGCGGTAAACCTTTGG	74	[27]
*S. ruminantium*	F:CAATAAGCATTCCGCCTGGGR:TTCACTCAATGTCAAGCCCTGG	138	[27]
Protozoa	F:GCTTTCGWTGGTAGTGTATTR:CTTGCCCTCYAATCGTWCT	223	[28]
Fungi	F:GAGGAAGTAAAAGTCGTAACAAGGTTTCR:CAAATTCACAAAGGGTAGGATGATT	120	[24]
Total bacteria	F:CGGCAACGAGCGCAACCCR:CCATTGTAGCACGTGTGTAGCC	146	[25]

**Table 3:** Effect of different gossypol addition levels on in vitro dry matter disappearance (IVDMD) and gas production kinetics of two rations after 48 h of incubation.

Item ^1^		IVDMD	GP_48_	Gas Production Kinetic				Gas Composition (%)		
				A	B	C	AGPR	CO_2_	CH_4_	H_2_
Ration	HF ^2^	53.5 ^b^	130.5	147.8	1.1	4.7	10.4 ^a^	83.0 ^a^	15.6	0.2
	LF ^2^	65.0 ^a^	118.6	131.7	1.1	4.7	8.0 ^b^	82.2 ^b^	16.3	0.2
SEM ^3^		0.50	4.17	7.94	0.03	0.56	0.78	0.20	0.20	0.01
Gossypol Dose (mg/g)	0	60.6	140.5 ^a^	156.3 ^a^	1.1 ^ab^	5.1	9.2	83.4 ^a^	15.4 ^b^	0.20 ^a^
	0.25	59.4	128.2 ^ab^	152.8 ^a^	1.0 ^b^	5.4	9.0	83.0 ^a^	16.0 ^ab^	0.18 ^ab^
	0.5	58.9	118.8 ^bc^	136.1 ^ab^	1.0 ^b^	4.9	8.0	82.0 ^b^	16.7 ^a^	0.16 ^b^
	0.75	58.6	111.8 ^c^	116.3 ^b^	1.2 ^a^	3.6	10.4	81.6 ^b^	15.8 ^b^	0.15 ^b^
SEM ^3^		0.70	4.62	9.71	0.03	0.75	1.17	0.30	0.20	0.01
*p*-Value	Ration	<0.01	0.02	0.17	0.13	0.96	0.04	0.04	0.06	0.81
	Dose	0.50	<0.01	0.05	0.02	0.39	0.36	<0.01	0.02	0.01
	I ^4^	0.90	0.58	0.75	0.28	0.56	0.27	0.90	0.04	0.80
	L ^4^	0.63	<0.01	<0.01	0.08	0.17	0.43	<0.01	0.18	<0.01
	Q ^4^	0.18	0.54	0.44	0.01	0.32	0.19	0.90	<0.01	0.40

^a,b,c^ Values in a column within the same class without a common superscript are significantly different (*p* < 0.05). ^1^ A, asymptotic gas production; B, sharpness parameter determining the curve shape of the cumulative gas production; C, the time (h) at which half of A is reached; AGPR, the average gas production rate at half of A; GP_48_, cumulative gas production at 48 h; IVDMD, in vitro dry matter disappearance. ^2^ HF, high-forage substrate; LF, low–forage substrate. ^3^ SEM, standard error of the difference of the means. ^4^ I, interaction effect; L, linear; Q, quadratic.

**Table 4:** Effect of different gossypol addition levels on in vitro fermentation characteristics of two rations after 48 h of incubation.

Item ^1^	Ration		SEM ^3^	Gossypol (mg/g)				SEM ^3^	*p*-Value				
	HF ^2^	LF ^2^		0	0.25	0.5	0.75		Ration	Dose	I ^4^	L ^4^	Q ^4^
pH	6.68 ^a^	6.61 ^b^	0.01	6.62 ^b^	6.64 ^ab^	6.66 ^ab^	6.67 ^a^	0.02	<0.01	0.09	0.99	0.01	0.87
NH_3-_N (mg/dL)	40.6 ^a^	37.3 ^b^	0.31	39.3	37.9	39.0	39.2	0.40	<0.01	0.22	0.05	0.91	0.07
MCP (μg/mL)	91.5	91.0	2.25	105.4 ^a^	91.1 ^b^	85.5 ^b^	83.0 ^b^	3.19	0.89	<0.01	0.68	<0.01	0.02
tVFA (mM)	93.1 ^a^	85.9 ^b^	1.09	89.6 ^ab^	90.8 ^a^	91.8 ^a^	85.8 ^b^	1.55	<0.01	0.06	0.53	0.15	0.03
Ace (%)	51.9 ^a^	47.2 ^b^	0.60	50.5 ^a^	49.9 ^a^	51.1 ^a^	46.9 ^b^	0.82	<0.01	0.01	0.15	0.01	0.03
Pro (%)	18.7 ^b^	19.7 ^a^	0.23	18.9 ^b^	20.3 ^a^	19.3 ^b^	18.5 ^b^	0.33	0.01	<0.01	0.23	0.10	<0.01
But (%)	13.0	13.0	0.16	12.4 ^b^	13.2 ^a^	13.5 ^a^	12.9 ^ab^	0.22	0.97	0.01	0.17	0.06	<0.01
Val (%)	2.1	2.0	0.03	2.0	2.1	2.1	2.1	0.05	0.14	0.93	0.09	0.55	0.83
BCVFA (%)	6.0 ^a^	5.3 ^b^	0.12	6.0 ^a^	5.0 ^b^	6.1 ^a^	5.6 ^a^	0.16	<0.01	<0.01	0.03	0.96	0.13
NGR	3.6	3.6	0.03	3.6 ^b^	3.5 ^b^	3.8 ^a^	3.6 ^b^	0.04	0.62	<0.01	0.77	0.21	0.58
FE	0.75	0.78	0.01	0.79	0.76	0.75	0.76	0.012	0.14	0.31	0.31	0.18	0.34
D_G_ (%)	97.6 ^a^	95.9 ^b^	0.12	-	96.8 ^b^	97.7 ^a^	95.8 ^c^	0.15	<0.01	<0.01	0.66	<0.01	<0.01

^a,b,c^ Values in a line within the same class without a common superscript are significantly different (*p* < 0.05). ^1^ tVFA, total VFA; Ace, acetate; Pro, propionate; But, butyrate; Val, valerate; BCVFA, branched-chain VFA; NGR, the ratio of non-glucogenic-to-glucogenic acids; FE, fermentation efficiency; D_G_: total disappearance of gossypol. ^2^ HF, high-forage substrate; LF, low-forage substrate. ^3^ SEM, standard error of the difference of the means. ^4^ I, interaction effect; L, linear; Q, quadratic.

**Table 5:** Effect of different gossypol addition levels on rumen microbial population of two rations after 48 h of incubation (log10 gene copy number/ mL of DNA extract).

Item	Ration		SEM ^2^	Gossypol (mg/g)				SEM ^2^	*p*-Value				
	HF ^1^	LF ^1^		0	0.25	0.5	0.75		Ration	Dose	I ^3^	L ^3^	Q ^3^
*F. succinogenes*	6.75 ^a^	6.50 ^b^	0.025	7.15 ^a^	6.34 ^d^	6.48 ^c^	6.61 ^b^	0.034	<0.01	<0.01	0.05	<0.01	<0.01
*R. flavefaciens*	6.78	6.68	0.080	6.67 ^b^	6.73 ^b^	6.41 ^b^	7.11 ^a^	0.110	0.41	<0.01	0.99	0.08	0.02
*R. albus*	6.32	6.25	0.021	6.42 ^a^	6.28 ^b^	6.24 ^b^	6.21 ^b^	0.026	0.07	<0.01	0.82	<0.01	0.11
*B. fibrisolvens*	8.50 ^b^	8.58 ^a^	0.016	8.72 ^a^	8.54 ^b^	8.49 ^b^	8.40 ^c^	0.022	<0.01	<0.01	0.60	<0.01	0.11
*P. ruminicola*	7.18 ^b^	7.26 ^a^	0.022	7.40 ^a^	7.01 ^d^	7.25 ^b^	7.14 ^c^	0.028	0.03	<0.01	0.02	<0.01	<0.01
*S. ruminantium*	6.09 ^b^	6.20 ^a^	0.028	6.23 ^a^	6.05 ^b^	6.15 ^ab^	6.15 ^ab^	0.034	0.01	0.06	0.62	0.51	0.06
Protozoa	4.97 ^a^	4.89 ^b^	0.016	4.76 ^c^	4.87 ^b^	4.94 ^b^	5.15 ^a^	0.022	<0.01	<0.01	0.82	<0.01	0.07
Fungi	3.36 ^a^	3.21 ^b^	0.016	3.45 ^a^	3.35 ^b^	3.26 ^c^	3.09 ^d^	0.023	<0.01	<0.01	0.31	<0.01	0.14
Total bacteria	9.82 ^b^	9.93 ^a^	0.035	9.78 ^b^	9.83 ^b^	9.86 ^b^	10.04 ^a^	0.049	<0.01	<0.01	<0.01	<0.01	0.04

^a,b,c,d^ Values in a line within the same class without a common superscript are significantly different (*p* < 0.05). ^1^ HF, high-forage substrate; LF, low-forage substrate. ^2^ SEM, standard error of the difference of the means.^3^ I, interaction effect; L, linear; Q, quadratic.

## Data Availability

The data presented in this study are available on request from the corresponding author.
